# What is the Role of Two-Dimensional Speckle Tracking Echocardiography in the Diagnosis and Management of Anthracycline-Induced Cardiotoxicity?

**DOI:** 10.5935/abc.20180047

**Published:** 2018-03

**Authors:** Isabela Bispo Santos da Silva Costa, Ludhmila Abrahão Hajjar

**Affiliations:** 1 Instituto do Câncer do Estado de São Paulo - Hospital das Clínicas da Faculdade de Medicina da Universidade de São Paulo (ICESP-HC-FMUSP), São Paulo, SP - Brazil; 2 Instituto do Coração do Hospital das Clínicas da Faculdade de Medicina da Universidade de São Paulo (INCOR - HC-FMUSP) São Paulo, SP - Brazil

**Keywords:** Neoplasms, Cardiotoxicity, Anthracyclines / toxicity, Ventricular Dysfunction, Troponin, Natriuretic Peptides, Echocardiography, Speckle-Tracking

The increasing number of patients with neoplasms and survivors^1,2^ has raised the interest of the scientific community in the diagnosis and early management of the effects of neoplasms and/or their treatments on patients. In that scenario, the injury caused to the cardiovascular system belongs to a spectrum and can impair all cardiovascular system structures in a clinically variable way, ranging from asymptomatic forms to cardiovascular death. Most studies on cardiotoxicity have focused on ventricular dysfunction because of its presentation severity and because it is the major cause of late non-oncologic mortality of neoplasm survivors.^3^

In oncologic patients, the drugs most commonly related to ventricular dysfunction are anthracyclines.^4^ Recent studies have reported that the damage related to those drugs, if not identified and treated early, evolves continuously from cell injury to ventricular dysfunction. In the past decade, several studies showed that the subclinical detection of cardiotoxicity, by use of biomarkers, such as troponin and BNP, might be an opportunity to prevent cardiovascular injury, allowing for early treatment and more appropriate individualized follow-up.^5-8^

Another current challenge regarding cardiotoxicity is to understand the natural history of neoplasm survivors. Little is known about the prevalence of cardiovascular disease in those patients, and, thus, no long-term follow-up strategy has been defined for them.

In this issue of the *Arquivos Brasileiros de Cardiologia*, Kang et al.^9^ make a relevant contribution to the diagnosis of anthracycline-induced cardiotoxicity. In a cohort of survivors of non-Hodgkin's diffuse large B cell lymphoma treated with anthracyclines, those authors have shown that, as compared to healthy controls, those patients have lower values of circumferential and longitudinal strains on echocardiography in a population with normal ejection fraction. Such findings have been evidenced mainly by changes in the subendocardial segments. In accordance with previous studies,^10^ those authors have emphasized the radial strain measure to be of little importance in that population. Inter- and intraobserver analyses reinforce that data obtained can be safely reproducible.

Kang et al.^9^ have not observed a direct relationship between anthracycline doses and strain values, suggesting that the myocardial damage, reflected on impaired myocardial deformation, can occur even at doses considered non-cardiotoxic (lower than 240 mg/m^2^), provided that the population studied used doses ranging from 150.94 mg/m^2^ to 440.00 mg/m^2^.

That was an observational study with a small sample, but its finding is clinically relevant and should be explored. It is yet to be defined whether that finding is only a marker of chemotherapeutic response or whether it represents the beginning of the pathophysiology of the clinically manifest cardiovascular lesion. Further studies are required to clarify whether the neoplasm itself, through its endothelial changes, could be related to changes in strain.

Even without definite responses, the study by Kang et al.^9^ contributes to reinforce the importance of combining the clinical practice with a sensitive non-invasive method to aid the management of oncologic patients during and after chemotherapy.^10,11^
